# Identifying D-Group Mitogen-Activated Protein Kinases as Substrates of *Arabidopsis* Tyrosine Phosphatase RLPH2 Using Phospho-Tyrosine Peptide Enrichment

**DOI:** 10.21769/BioProtoc.5810

**Published:** 2026-09-05

**Authors:** Anne-Marie Labandera, Brooklyn Kurucz, R. Glen Uhrig, Greg B. Moorhead

**Affiliations:** 1 INRAE, ONF, UMR BioForA, Orléans, France; 2 Department of Biological Sciences, University of Calgary, Calgary, AB, Canada; 3 Department of Biological Sciences, University of Alberta, Edmonton, AB, Canada; 4 Department of Biochemistry, University of Alberta, Edmonton, AB, Canada

**Keywords:** Phospho-proteomics, Tyrosine phosphatase, Mitogen-activated protein kinase, *Arabidopsis thaliana*, Protein kinase activation loop, Quantitative mass spectrometry

## Abstract

Identifying substrates of protein phosphatases has been technically challenging and has hampered progress in the field of plant sciences. Small molecule inhibitors of protein phosphatases have aided in uncovering classes of phosphatases that target substrates, but that too has severe limitations. Here, we describe a method that enriches phosphorylated substrates using TiO_2_ and phospho-tyrosine antibodies in phosphatase knockout lines of *Arabidopsis thaliana*. When compared to wild-type plants, this approach permits identification of putative substrates and specific phosphorylation sites by mass spectrometry, allowing for further in vitro or functional validation. The key to the approach described here is the use of phosphatase knockout lines to maintain substrates in a phosphorylated state and using phospho-tyrosine antibodies to enrich for tyrosine phosphorylated peptides.

Key features

• Requires genetic knockout lines for the protein phosphatase of interest to uncover increased phosphorylation of putative substrates compared to wild type.

• Phospho-peptides are (quantitatively) compared using mass spectrometry.

• Tyrosine phosphorylated peptides are immunoprecipitated after TiO_2_ phospho-peptide enrichment.

• Putative substrates identified with this protocol can later be validated with in vitro assays using synthetic phospho-peptides and/or phospho-proteins.

## Graphical overview


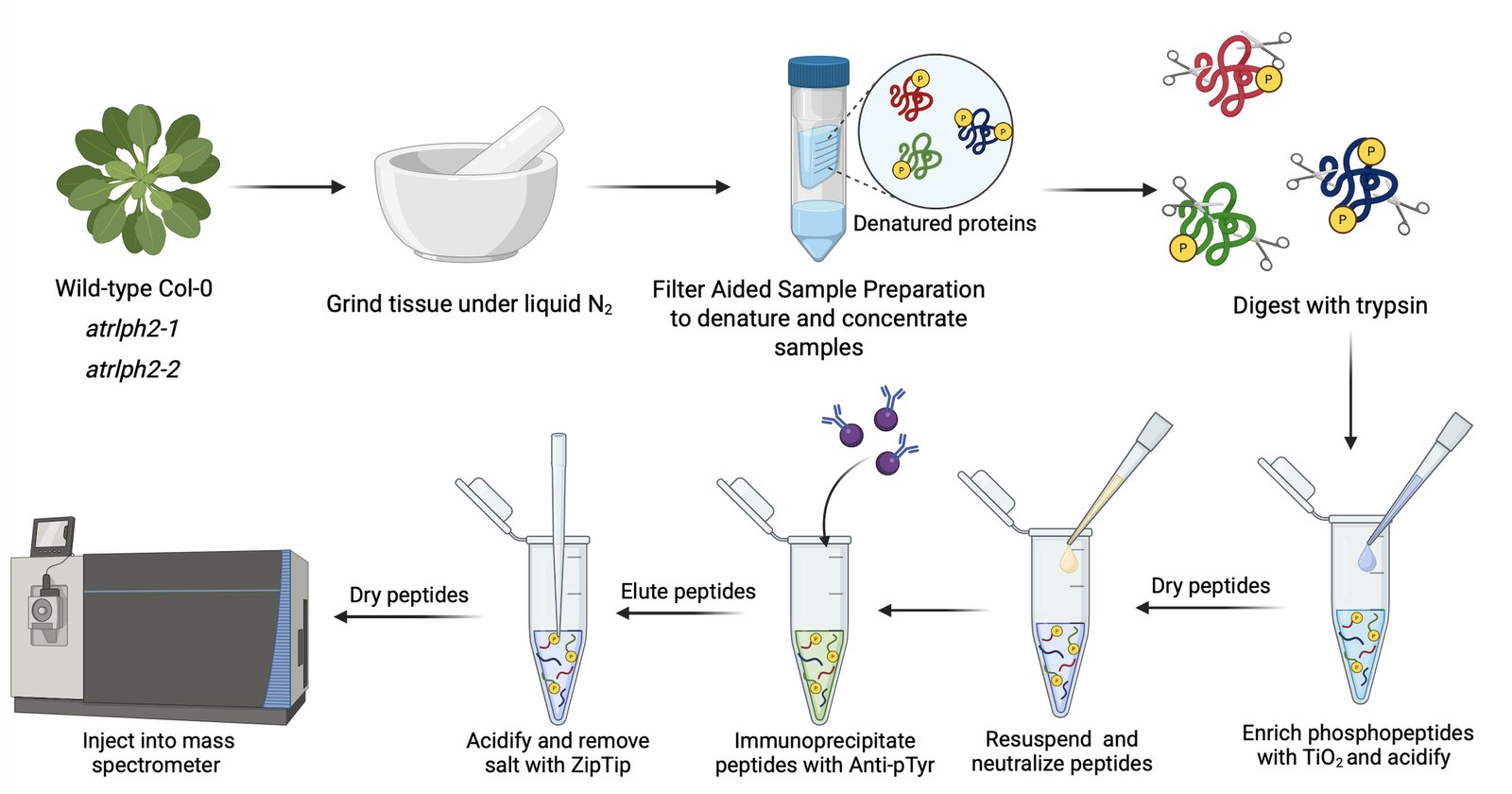


**Schematic of the RLPH2 phospho-tyrosine (pY) substrate enrichment pipeline**

## Background

Protein post-translational modifications (PTMs) such as phosphorylation largely regulate cellular signaling pathways by changing the protein’s structural properties, its subcellular localization, its binding capacity to other proteins, or its activity [1]. In plants, protein phosphorylation has been linked to diverse functions such as plant growth and development, defense, cell cycle regulation, and hormone biosynthesis and signaling [2]; therefore, it is crucial to properly characterize the players involved in this process.

Protein kinases add a phosphoryl group principally onto serine (Ser), threonine (Thr), and tyrosine (Tyr), whereas protein phosphatases remove phosphate. Large-scale plant phospho-proteomics has shown that the abundance of phospho-serine, phospho-threonine, and phospho-tyrosine in plants parallels that in humans, namely 84%–86%, 10%–12%, and 2%–4%, respectively [3,4]. Although levels of tyrosine phosphorylation are similar in plants and humans, there are very few tyrosine-specific phosphatases identified to date [5,6].

Identifying the substrates of protein phosphatases has been technically challenging, and progress in understanding these enzymes has fallen behind knowledge of the counteracting enzymes, the protein kinases. Recently, we have identified a novel type of protein phosphatase [7,8], the *Arabidopsis thaliana Rhizobiales*–like phosphatase 2 (RLPH2). It was predicted to be a serine/threonine-specific phosphatase due to its primary sequence; however, in vitro phospho-peptide assays revealed its preference for phospho-tyrosine residues. This was later supported by the crystal structure that demonstrated a deeper active site pocket than other protein phosphatases of the same family (the PPP-family); therefore, the RLPH2 active site is able to accommodate the larger phospho-tyrosine substrates [9].

Mass spectrometry analysis of covalent modifications has completely altered our view of the extent of these key regulatory features of proteins, particularly their prevalence in vivo. This is true not only for protein phosphorylation but also for other post-translational modifications, including methylation, acetylation, and glycosylation [10,11]. The sensitivity of this approach is contingent upon enriching covalently modified peptides and has allowed the discovery of low-abundance sites. For phospho-peptides, this typically requires the use of TiO_2_ beads that will bind modified phospho-peptides to serine, threonine, and tyrosine [12,13]. Phospho-tyrosine typically represents only 2% of a eukaryotic phosphoproteome; thus, we have employed anti-phospho-tyrosine antibodies coupled to a Sepharose/agarose matrix to further enrich for this modification that could be masked by phospho-serine/threonine peptides.

In vitro work has revealed the substrate specificity of RLPH2 and hinted at the important structural features the substrates should possess [7]. To identify endogenous substrates, T-DNA knockout (KO) lines of the RLPH2 phosphatase were used along with their corresponding wild-type control. This approach can also be used in any organism with the protein phosphatase knocked out or edited by CRISPR-Cas technology to express a non-active phosphatase. When coupled with phospho-peptide enrichment and mass spectrometry, the door opens to decipher the substrates of protein phosphatases of all classes.

## Materials and reagents

**Biological materials**

1. *Arabidopsis thaliana* Nössen wild type

2. T-DNA insertion mutant lines for protein phosphatase RLPH2 (AT3G09970), *atrlph2-1*, and *atrlph2-2* in Nössen background; seeds purchased from RIKEN, *atrlph2-1* (RATM-13-2130-1_G), and *atrlph2-2* (RATM-13-3204-1_G)

**Reagents**

1. Murashige and Skoog medium (MS) (Phytotech Labs, catalog number: M401)

2. Agar (Sigma-Aldrich, CAS No.: 9002-18-0)

3. HEPES (Sigma-Aldrich, CAS No.: 7365-45-9)

4. SDS (Sigma-Aldrich, CAS No.: 151-21-3)

5. DTT (Thermo Scientific, catalog number: 20290)

6. Coomassie Brilliant Blue G-250 (Bio-Rad, catalog number: 1610406)

7. 95% ethanol (Fisher Scientific, CAS No.: 64-17-5)

8. 85% phosphoric acid (Sigma-Aldrich, CAS No.: 7664-38-2)

9. Tris (Sigma-Aldrich, CAS No.: 77-86-1)

10. Urea (Sigma-Aldrich, CAS No.: 57-13-6)

11. EDTA (Sigma-Aldrich, CAS No.: 60-00-4)

12. Iodoacetamide (Sigma-Aldrich, CAS No.: 144-48-9)

13. Ammonium bicarbonate (Sigma-Aldrich, CAS No.: 1066-33-7)

14. Trypsin (Promega, catalog number: V5113)

15. NaCl (Sigma-Aldrich, CAS No.: 7647-14-5)

16. Trifluoracetic acid (Sigma-Aldrich, CAS No.: 76-05-1)

17. Acetonitrile (Sigma-Aldrich, CAS No.: 75-05-8)

18. Formic acid (FA) (Sigma-Aldrich, CAS No.: 64-18-6)

19. Ammonium hydroxide (Sigma-Aldrich, CAS No.: 1336-21-6)

20. Sodium hydroxide (Sigma-Aldrich, CAS No.: 1310-73-2)

21. TiO_2_ beads (Merck, CAS No.: 13463-67-7)

22. Anti-phospho-tyrosine antibody agarose beads (Santa Cruz, catalog number: sc-7020)

23. Anti-phospho-tyrosine antibody Sepharose beads (Cell Signaling, catalog number: 7902, p-Tyr-100)

**Solutions**

1. 0.5× MS-agar (see Recipes)

2. Extraction buffer (see Recipes)

3. Bradford reagent (see Recipes)

4. Urea buffer (see Recipes)

5. IAA solution (see Recipes)

6. IAA urea solution (see Recipes)

7. ABC buffer (see Recipes)

8. ABC buffer with trypsin (see Recipes)

9. Salt solution (see Recipes)

10. 25% (v/v) TFA (see Recipes)

11. Desalt binding buffer (see Recipes)

12. Desalt elution buffer (see Recipes)

13. 30% ACN solution, 0.1% TFA (see Recipes)

14. Loading buffer (see Recipes)

15. 20% (v/v) formic acid (FA) (see Recipes)

16. Wash buffer (see Recipes)

17. 70% EtOH (see Recipes)

18. Elution buffer 1 (see Recipes)

19. Elution buffer 2 (see Recipes)

20. IP buffer (see Recipes)

21. 0.1 M NaOH (see Recipes)

22. IP elution buffer (see Recipes)

23. Solvent A (see Recipes)

24. Solvent B (see Recipes)

**Recipes**

**1. 0.5× MS-agar, pH 5.7**

**Table:** 

Reagent	Final concentration	Quantity or volume
MS	0.5×	2.2 g
Agar	0.6 %	6 g
ddH_2_O	n/a	1 L
Total	n/a	1 L

**2. Extraction buffer, pH 8.0**

**Table:** 

Reagent	Final concentration	Quantity or volume
HEPES	25 mM	595.75 mg
SDS	4%	4 g
DTT	100 mM	1.54 g
ddH_2_O	n/a	100 mL
Total	n/a	100 mL

**3. Bradford reagent**

**Table:** 

Reagent	Final concentration	Quantity or volume
Coomassie Brilliant Blue G-250	0.1% (w/v)	100 mg
95% ethanol	4.7% (w/v)	50 mL
85% phosphoric acid	8.5% (w/v)	100 mL
ddH_2_O	n/a	850 mL
Total	n/a	1 L

**4. Urea buffer, pH 8.0**

**Table:** 

Reagent	Final concentration	Quantity or volume
Tris	50 mM	302.85 mg
Urea	8.0 M	24.02 g
EDTA	2 mM	29.22 mg
ddH_2_O	n/a	50 mL
Total	n/a	50 mL

**5. IAA solution**

**Table:** 

Reagent	Final concentration	Quantity or volume
Iodoacetamide	500 mM	92.48 g
ddH_2_O	n/a	1 L
Total	500 mM	1 L

**6. IAA urea solution**

**Table:** 

Reagent	Final concentration	Quantity or volume
500 mM iodoacetamide	50 mM	100 mL
Urea buffer (Recipe 4)	n/a	900 mL
Total	50 mM	1 L

**7. ABC buffer, pH 8.5**

**Table:** 

Reagent	Final concentration	Quantity or volume
Ammonium bicarbonate	50 mM	98.82 mg
ddH_2_O	n/a	25 mL
Total	50 mM	25 mL

**8. ABC buffer with trypsin at 1:100 (enzyme: substrate)**

**Table:** 

Reagent	Final concentration	Quantity or volume
Ammonium bicarbonate buffer (Recipe 7)	50 mM	9.90 mL
Trypsin	1:100 (trypsin:peptide)	100 μL
Total	n/a	10 mL

**9. Salt solution**

**Table:** 

Reagent	Final concentration	Quantity or volume
NaCl	500 mM	29.22 g
ddH_2_O	n/a	1 L
Total	500 mM	1 L

**10. 25% (v/v) TFA**

**Table:** 

Reagent	Final concentration	Quantity or volume
Trifluoracetic acid	25% (v/v)	25 mL
ddH_2_O	n/a	75 mL
Total	25% (v/v)	100 mL

**11. Desalt binding buffer**

**Table:** 

Reagent	Final concentration	Quantity or volume
Acetonitrile	3% (v/v)	3.0 mL
25% (v/v) TFA (Recipe 10)	0.1% (v/v)	400 μL
ddH_2_O	n/a	96.6 mL
Total	n/a	100 mL

**12. Desalt elution buffer**

**Table:** 

Reagent	Final concentration	Quantity or volume
Acetonitrile	60% (v/v)	60 mL
25% (v/v) TFA (Recipe 10)	0.1% (v/v)	400 μL
ddH_2_O	n/a	39.6 mL
Total	n/a	100 mL

**13. 30% ACN solution, 0.1% TFA**

**Table:** 

Reagent	Final concentration	Quantity or volume
Acetonitrile	30% (v/v)	30 mL
Trifluoracetic acid	0.1% (v/v)	100 μL
ddH_2_O	n/a	69.9 mL
Total	n/a	100 mL

**14. Loading buffer**

**Table:** 

Reagent	Final concentration	Quantity or volume
Acetonitrile	80% (v/v)	80 mL
Trifluoracetic acid	6% (v/v)	6 mL
ddH_2_O	n/a	14 mL
Total	n/a	100 mL

**15. 20% (v/v) FA**

**Table:** 

Reagent	Final concentration	Quantity or volume
FA	20% (v/v)	20 mL
ddH_2_O	n/a	80 mL
Total	20% (v/v)	100 mL

**16. Wash buffer**

**Table:** 

Reagent	Final concentration	Quantity or volume
Acetonitrile	50% (v/v)	50 mL
Trifluoracetic acid	0.1% (v/v)	100 μL
ddH_2_O	n/a	49.9 mL
Total	n/a	100 mL

**17. 70% EtOH**

**Table:** 

Reagent	Final concentration	Quantity or volume
95 % ethanol	70% (v/v)	73.68 mL
ddH_2_O	n/a	26.32 mL
Total	70% (v/v)	100 mL

**18. Elution buffer 1, pH 11.0**

**Table:** 

Reagent	Final concentration	Quantity or volume
Ammonium hydroxide	5% (v/v)	95 mL
ddH_2_O	n/a	5 mL
Total	5% (v/v)	100 mL

**19. Elution buffer 2**

**Table:** 

Reagent	Final concentration	Quantity or volume
Acetonitrile	80% (v/v)	80 mL
FA	2% (v/v)	2 mL
ddH_2_O	n/a	18 mL
Total	n/a	100 mL

**20. IP buffer, pH 7.4**

**Table:** 

Reagent	Final concentration	Quantity or volume
HEPES	50 mM	1.19 g
NaCl	50 mM	292.2 mg
ddH_2_O	n/a	100 mL
Total	n/a	100 mL

**21. NaOH**

**Table:** 

Reagent	Final concentration	Quantity or volume
NaOH	0.1 M	4.00 g
ddH_2_O	n/a	1 L
Total	0.1 M	1 L

**22. IP elution buffer**

**Table:** 

Reagent	Final concentration	Quantity or volume
Acetonitrile	80% (v/v)	80 mL
FA	2% (v/v)	2 mL
ddH_2_O	n/a	18 mL
Total	n/a	100 mL

**23. Solvent A**

**Table:** 

Reagent	Final concentration	Quantity or volume
FA	0.1% (v/v)	100 μL
ddH_2_O	n/a	99.9 mL
Total	0.1% (v/v)	100 mL

**24. Solvent B**

**Table:** 

Reagent	Final concentration	Quantity or volume
Acetonitrile	100% (v/v)	99.9 mL
FA	0.1% (v/v)	100 μL
Total	0.1% (v/v)	100 mL

**Laboratory supplies**

1. Sep-Pak C18 cartridges (Waters, catalog number: WAT054960)

2. C8 stage tips 200 μL (Thermo Scientific, catalog number: SP321)

3. Amicon Ultra Centrifugal Filters (centricons) MW cutoff 30 kDa (Millipore, catalog number: UFC901096)

4. ZipTip C18 pipette tips (Millipore, catalog number: ZTC18S960)

5. Mortar and pestle (CoosTek, catalog number: 60332)

6. Falcon tubes (15 and 50 mL) (Millipore, catalog numbers: CLS352096, CLS352070)

7. pH indicator strip (MicroEssentialLab, catalog number: F01-WIDRG-010140-SRD)

## Equipment

1. Speed vacuum, SpeedVac SPD210 vacuum concentrator (Thermo Fisher, catalog number: SPD210-230)

2. Centrifuge swing rotor for Falcon tubes 50 mL, TX-150 Swinging Bucket Rotor, compatible with Thermo Scientific ST8 Megafuge 8 and SL8 centrifuges (Thermo Fisher, catalog number: 750055701)

3. Microtube centrifuge, Accuspin Micro 17/17R Microcentrifuge (Fisher Scientific, catalog number: 13100675)

4. C18 nano-flow analytical column, nanoEase M/Z Peptide BEH C18 Column 130 Å, 1.7 μm, 300 μm × 150 mm (Waters, catalog number: 186009259)

5. Nanoflow LC (nLC1200) (Thermo Fisher), Nanoflow LC (nLC1200) (Thermo Fisher, catalog number: ES803)

6. High-resolution mass spectrometer (Thermo Fisher or equivalent), Orbitrap Fusion Mass Spectrometer (Thermo Fisher, catalog number: FETD2-10002)

7. Tabletop Eppendorf shaker, Orbi-Shaker MP (Sigma-Aldrich, catalog number: Z742450)

8. Heating block, Corning LSE Single Block (Sigma-Aldrich, catalog number: CLS480124-1EA)

9. Vortex, Corning LSE Vortex Mixtures (Sigma-Aldrich, catalog number: CLS6775-1EA)

10. Spectrophotometer or Nanodrop (capable of measuring 280 nm), Nanodrop One/One Microvolume UV-Vis Spectrophotometer (Thermo Fisher, model: ND-ONE-W)

11. End-over-End Rotator, Benchmark RotoMini Rotator (Sigma-Aldrich, model: BMSR2020-1EA)

## Procedure

**A. Plant growth and harvesting**

1. Surface-sterilize seeds from *A. thaliana* wild type (WT) as well as *atrlph2-1* and *atrlph2-2* mutant lines for 4 h in sterilization solution and plate on 0.5× MS agar plates.

2. Stratify in the dark at 4 °C for 2 days. Place plates under constant light for 10 days.

3. Transfer the seedlings onto soil and grow in 12/12 h light/dark conditions for 25 days (harvested prior to bolting).

4. Harvest rosettes during the light period, flash-freeze them in liquid N_2_, and grind with a mortar and pestle under liquid N_2_.

5. Store rosette powder at -80 °C until the day of use.

**B. Filter-aided sample preparation (FASP)**

**B1. Extraction and digestion**

1. Add 1 mL of extraction buffer per 0.6 g of tissue powder, grind in a mortar with pestle, and boil for 5 min at 95 °C.

2. Incubate horizontally on a tabletop shaker at 300 rpm for 40 min at room temperature (RT) while quickly vortexing every 10 min.

3. Centrifuge at maximum speed for 15 min at RT and keep the supernatant.

4. (**KEY STEP**) Measure protein extract concentration using Bradford reagent [14] blanked with extraction buffer. All samples and blank must be diluted in ddH_2_O to a 1:5 ratio.

5. Transfer 10–12 mg (25 mg max) of total protein to a fresh 15 mL Falcon tube.

6. Condition the centricon/Ultra Centrifugal Filter membrane (30 kDa MWCO) with 5 mL of urea buffer using a swing bucket centrifuge. Centrifuge at 4,000× *g* for 10 min.

7. Add 15 mL of 8 M urea buffer to the extracted proteins (the SDS from the extraction buffer must be diluted to 0.5% or less).

8. Centrifuge at 4000× *g* for 15 min or until a 10-fold concentration (1.5 mL total final volume).

9. Repeat steps A7–8.

10. Add 3 mL of IAA urea solution and gently mix by pipetting up and down.

11. Incubate in the dark at RT for 30 min without shaking.

12. Centrifuge at 4,000× *g* for 10 min.

13. Wash by adding 15 mL of urea buffer and centrifuge at 4,000× *g* for 45 min.

14. Add 15 mL of ABC buffer and centrifuge at 4,000× *g* for 18 min.

15. Transfer the centricon/Ultra Centrifugal filter to a fresh 50 mL conical tube and keep the original lid.

16. Add 3 mL of ABC buffer with trypsin at 1:100 (enzyme:substrate) and leave shaking overnight at 37 °C and 150 rpm on a 35° angle.

**B2. Peptide isolation**

1. Centrifuge the trypsinized solution (still in the centricon/Ultra Centrifugal Filter) at 4,000× *g* for 15 min and keep the flowthrough.

2. Gently pipette the remaining liquid up and down on the membrane to dislodge any debris from the filter and repeat step B2.1.

3. Add 3 mL of ABC buffer and centrifuge at 4,000× *g* for 20 min.

4. Add 3 mL of 500 mM NaCl and centrifuge at 4,000× *g* for 45 min.

5. Acidify the solution with 25% (v/v) TFA to a final concentration of 0.5% TFA.

**B3. Peptide desalting**

1. Wash the Sep-Pak Light C18 cartridges with 1 mL of methanol. Samples and solutions can be loaded and pushed gently through the cartridge using a 2 mL syringe.

2. Wash with 1 mL of desalt elution buffer.

3. Equilibrate the membrane with 3 × 1 mL of desalt binding buffer.

4. Load peptide sample.

5. Wash twice with 1 mL of desalt binding buffer.

6. Elute desalted peptides with 2 × 500 μL of desalt elution buffer into microcentrifuge tubes.

**C. Phospho-peptide enrichment with TiO_2_ beads**

1. Resuspend 10 mg of TiO_2_ beads in 1 mL of 30% ACN solution, 0.1% TFA.

2. Use 50 μL of the slurry (10 mg/mL) with 1 mL of loading buffer for each affinity enrichment.

3. Pellet TiO_2_ at 1,000× *g* for 1 min and remove the supernatant without disturbing the beads.

4. Add total peptide solution to the pelleted TiO_2_ and incubate end-over-end for 45 min at RT.

5. Pierce the lids of 2 mL Eppendorf tubes and mount in the C8 stage tips.

6. Wash with 100 μL of 100% methanol and spin at 1,000× *g* for 2 min.

7. Wash with 100 μL of loading buffer.

8. Pellet peptide-coupled matrix at 1,000× *g* for 5 min.

9. (**KEY STEP**) If used for quantification, keep the supernatant and dry non-phosphorylated peptides with SpeedVac.

10. Resuspend TiO_2_ pellet in 100 μL of loading buffer.

11. Load C8 tip and spin at 1,000× *g* for 5 min. Discard the flowthrough.

12. Wash with 50 μL of loading buffer and spin at 1,000× *g* for 10 min.

13. Wash with 100 μL of wash buffer and spin at 1,000× *g* for 10 min. Repeat this step.

14. Wipe the tip with a 70% EtOH wet Kimwipe and place the tip in a fresh 1.5 mL Eppendorf tube containing 50 μL of 20% FA.

15. Add 50 μL of elution buffer 1 and spin at 1,000× *g* for 5 min. Repeat this step.

16. Add 100 μL of elution buffer 2 and spin at 1,000× *g* for 10 min.

17. Acidify the eluted sample with 5 μL of 100% (v/v) FA; ensure it is acidified.

*Note: This step is important due to the basic pH of the ammonium hydroxide (pH 11.0).*

18. Check pH with pH indicator strip. If necessary, adjust with 20% (v/v) FA.

19. Dry each sample with SpeedVac.

**D. Phospho-tyrosine peptide isolation by immunoprecipitation**

1. Resuspend phospho-peptides in 800 μL of ice-cold IP buffer.

2. Mix and leave for 20 min at RT and pulse vortex 5 × 3 s. Ensure that all peptides are in solution.

3. Adjust pH to approximately 7.5 with NaOH using a pH indicator strip.

4. Add 50 μL of anti-phospho-tyrosine antibody agarose beads and 15 μL of anti-phospho-tyrosine antibody Sepharose beads (p-Tyr-100) to an Eppendorf tube, gently pellet, add 500 μL of IP buffer, gently mix, and pellet beads again.

5. Add 500 μL of IP buffer to resuspend beads.

6. Add phospho-peptides and incubate end-over-end overnight at 4 °C.

7. Gently pellet beads by spinning at 1,000× *g* for 1 min.

*Note: Dry supernatant with SpeedVac and freeze at -20 °C for later analysis to confirm that prior phospho-peptide enrichment with TiO_2_ worked well.*

8. Wash three times with 1 mL of IP buffer end-over-end for 2 min at RT. Gently pellet beads, remove supernatant, and repeat wash.

9. Wash three times (as in step D8) with 1 mL of ddH_2_O end-over-end for 2 min at RT.

10. Add 200 μL of IP elution buffer, mix gently, pellet beads, and keep eluted peptides. Repeat two more times and pool the three eluted 200 μL of peptides in IP elution buffer.

11. Dry with SpeedVac.

**E. Mass spectrometry analysis**

1. Dissolve phospho-tyrosine peptides in 3% ACN (v/v)/0.1% (v/v) TFA.

2. To desalt the phospho-tyrosine peptides, equilibrate ZipTip C18 pipette tips by loading 3% ACN (v/v)/0.1% (v/v) TFA onto the tip previously placed into a tip adaptor or pierced microcentrifuge tube.

3. Bind the acidified phospho-tyrosine peptides by loading them onto the ZipTip C18 tips.

4. Wash three times with 3% ACN (v/v)/0.1% (v/v) TFA and elute into a fresh tube using desalt elution buffer.

5. Dry in SpeedVac and dissolve in 3.0% ACN (v/v)/0.1% FA (v/v).

6. Prepare a 50 cm EasySpray nano-LC column (ES803) by equilibrating it with 100% solvent A (0.1% FA in water).

7. Inject samples into an Easy-nLC 1000 system and separate the peptides on the previously equilibrated column.

8. Elute using the following gradient of solvent B (0.1% FA in ACN): 0–50 min; 0%–25% B, 50–60 min; 25%–32% B, 60–70 min; 32%–98% B at a flow rate of 0.3 μL/min.

9. Acquire high-accuracy mass spectra with a high-resolution mass spectrometer operated in data-dependent acquisition (DDA) mode.

*Note: Many mass spectrometer manufacturers have instrumentation for acquiring DDA. These include Thermo Fisher Scientific (e.g., Orbitrap/Astral), Bruker (e.g., TimsTOF), and SCIEX (e.g., ZenoTOF). The details of how to acquire high-resolution DDA data for phospho-tyrosine peptides should be discussed with the specific manufacturer or a proteomics facility.*

## Data analysis

Data analysis can be performed using any software that accommodates acquired data dependent acquisition (DDA) mass spectrometry data that also allows for post-translational site modification identification. Examples of relevant software include MaxQuant (https://maxquant.org/), FragPipe (https://fragpipe.nesvilab.org/), and Scaffold (https://www.proteomesoftware.com/), amongst others. Relevant statistical thresholds when searching the data should include protein, peptide, and PSM false discovery rate (FDR) of 1%, along with variable modifications set to include phosphorylated tyrosine. Downstream data analysis should include a relevant corrected p-value (ideally less than 5%) for larger systemic studies comparing conditions. Generally, biological replication (n = 4), where possible, is ideal for robust quantitative experimentation.

## Validation of protocol

This protocol (or parts of it) has been used and validated in the following research article(s):

Labandera et al. [9]. Phospho-Proteomics Identifies D-Group MAP Kinases as Substrates of the Arabidopsis Tyrosine Phosphatase RLPH2. Plant Direct (Figure 3). DOI: 10.1002/pld3.70137

## General notes and troubleshooting

**General notes**

1. Although the *Arabidopsis thaliana* Nössen ecotype is used here, any ecotype is appropriate depending on the availability of the desired knockout line. It is always necessary to confirm knockout lines first with PCR analysis and, if possible, by western blot.

2. It is crucial that the tissue is harvested always at the same time of the day for all repetitions because the phosphoproteome fluctuates across photoperiod transitions, which can generate inconsistent results [15].

3. To calculate the trypsinization efficiency, measure peptide concentration by absorbance at 280 nm with an assumption that 1.0 absorption equals 1.0 mg/mL of peptide. Calculate trypsinization efficiency related to starting protein concentration. Efficiency should be at least 50%–60%.

4. In the literature, both anti-phospho-tyrosine antibodies are regarded as excellent; we chose to use both to potentially increase the outcome of binding all phospho-tyrosine-containing peptides. The TiO_2_ beads and the anti-phospho-tyrosine Sepharose/agarose matrices are easily pelleted with a spin at 1,000× *g* for 1 min. Care must be taken not to spin for longer or at a higher g-force because the matrix can be crushed and generate fine particles when Sepharose/agarose matrices are employed.

**Troubleshooting**

1. When conditioning the centricons, it is important to check that the flow is not too fast or too slow. Not all filters will behave the same way; therefore, centricons that do not have a satisfactory flow should be discarded before continuing with the protocol.

2. For first-time users, it might be difficult to understand how to use the C8 stage tips and C18 Zip Tips. The tips are inserted into the 2 mL microcentrifuge tube and loaded from the top, never from the bottom. These tips are used as gravity columns. A detailed description of the use of the C8 stage tips (step B3) and C18 Zip Tips (section E) is found on the manufacturer’s website.

3. If the general phospho-peptide yield is low (see step D8), it might be due to upstream issues in the protocol. For instance, it could be due to centricon leakage. Although more expensive, the use of new centricons each time is highly recommended. To avoid loss of sample, the peptide digestion needs to be at an angle of 35° from the vertical position. If the angle is too vertical, there will be improper mixing.
